# Effectiveness of the Electromagnetic Shock Wave Therapy in the Treatment of Cellulite

**DOI:** 10.1155/2019/8246815

**Published:** 2019-06-20

**Authors:** Débora Apª O. Modena, Caroline Nogueira da Silva, Talita C. P. Delinocente, Tatiane Bianca de Araújo, Tania Maria de Carvalho, Clovis Grecco, Renata Gomes Moreira, Gisele Campos, José Ricardo de Souza, Renata Michelini Guidi

**Affiliations:** ^1^State University of Campinas (Unicamp), Campinas, SP, Brazil; ^2^Ibramed Research Group: Study Group of Technology Applications in Health, IBRAMED, Amparo, SP, Brazil; ^3^University Center of Jaguariúna (UNIFAJ), Brazil; ^4^Center for Study and Advanced Training Ibramed (CEFAI), Amparo, SP, Brazil

## Abstract

In the past centuries, the human body was undervalued; nowadays, however, it is overvalued, and thus the manifestation of the dissatisfactions regarding the body has been increasing. Most of the time, these dissatisfactions are related to cellulite, which is most common in women. Its treatment is one of the challenges which encourage the development of new therapeutic modalities, among them the shockwave therapy.* Objective*. To evaluate the efficacy of ESWT in the treatment of cellulite in gluteus and posterior of thigh.* Method*. This is a prospective and comparative study, in which volunteer women who attended the criteria of inclusion were selected and who were subjected to 10 sessions of ESWT. The following were performed as an evaluation method: anthropometry, perimetry, skin viscoelasticity with the Cutometer®, thickness of hypodermis with diagnostic ultrasound, analysis of the scale of severity of cellulite (CSS), and quality of life by the Celluqol® questionnaire. The evaluations occurred before the first session (baseline), after 6 and 10 sessions, and during a follow-up of 3 months after the last session. The statistical test applied was the ANOVA one-way with post hoc of Tukey (P-value < 0.05).* Results*. There was significant improvement (P<0.05) for CSS, for the variable referring to gross elasticity and skin deformation ability evaluated in the Cutometer® and improvement of quality of life represented by Celluqol®. The result was maintained particularly in the follow-up of 3 months after the end of the treatment.* Conclusion*. The results presented demonstrated the effectiveness and safety of ESWT in the treatment of cellulite and in the decrease of the degrees, improvement of the aspect of the skin, and reestablishment of quality of life. This trial is registered with ClinicalTrials: NCT03275259.

## 1. Introduction

Nowadays, the search for the ideal body is related to internal dissatisfactions that generate low self-esteem. In the last decades the body was undervalued; today, it is overvalued and has become a valuable good [1]. For that reason, the search for invasive or noninvasive treatments in esthetic medicine has been increasing alarmingly [1, 2].

One of the most common dissatisfactions regarding the body which produces complaints among women after puberty is cellulite, also known as gynoid lipodystrophy and edematous fibrosclerotic panniculopathy [3–5].

This is a chronic esthetic condition which affects mainly gluteus and thighs and stems from metabolic and anatomical disorders of the skin and subcutaneous tissue, with visible alterations in the appearance of the body contour, caused by the damage to the fibrotic septum, generating deformities such as depression and elevation of the skin relief commonly referred to as “orange peel” or “cottage cheese” [3–6].

The cellulite diagnosis is carried out using some evaluation methods, which are not clearly defined. In 1978, Nurnberger and Müller developed a method of clinical evaluation for cellulite which characterizes it in degrees of severity between degrees zero, one, two, and three [7]. The scale is used as a reference evaluation for esthetic treatments; however, it presents limitations because it does not evaluate the morphological aspects which affect the severity of cellulite [7].

A new scale of evaluation of the severity of cellulite was proposed by Hexsel et al., 2009, which consisted of the evaluation of the aspects of skin texture, such as the number and depth of depressions, classification of the morphological appearance of the skin, degrees of flaccidity, and also the scale of Nurnberger and Müller. The evaluator performs the sum of the score of each item and classifies the cellulite between light degree (1-5 points), moderate (6-10 points), and severe (11-15 points). According to the authors, the tool influences the conduction and result of the treatment [8, 9].

A large number of therapeutic modalities for the treatment of cellulite have been proposed; among them are topic agents such as cosmetics, ultrasound, radiofrequency laser therapy, therapeutic massage, vibration/oscillation platform therapy, carboxytherapy, intense pulsed light, and most recently extracorporeal shockwave therapy (ESWT) [10–12].

According to Modena et al., 2017, ESWT began to be used in esthetic treatments after observations of the use of this therapy in orthopedic treatments, in which it became evident, besides the improvement in mobility and pain and the improvement of the aspect of skin and body contour, thus suggesting that there was a decrease in body circumference in the treated region [9, 13].

In literature, about twelve scientific articles were found about the subject, which demonstrate the efficacy of shockwave therapy as a safe noninvasive method. It is still not an expressive number about the technique and there is a demand for further investigation to obtain more reliability in the results of the therapy, as well as demonstrate the importance of the evaluation of the psychological state of the patient regarding self-esteem and quality of life of women who suffer from this esthetic disorder and who undergo the treatment with ESWT [13].

In the present study, the objective was to evaluate the effect of shockwave therapy in patients with different degrees of severity in the gluteal and posterior thigh regions. Anthropometric parameters, thickness of the adipose layer, and skin elasticity were evaluated and a questionnaire was applied to evaluate the quality of life before and after shockwave therapy.

## 2. Methodology

This study presents a comparative and prospective longitudinal clinical study, approved by the local Committee of Ethics in Research and registered under ClinicalTrials: NCT03275259. All the individuals were recruited at the Center for Advanced Studies (Centro de Estudos e Formação Avançada) IBRAMED (CEFAI), were informed about the procedure, and signed the prior informed consent form.

The main eligibility criteria were women between 18 and 45 years of ages, with body mass index (BMI) of up to 29.9; nonsmokers; without previous disorders; with light, moderate, and severe degrees of cellulite according to the Cellulite Severity Scale (CSS) [8] in the gluteal and posterior thigh regions. The main criteria of ineligibility were participants with other treatments for cellulite less than 30 days before the study, suffering from dermatitis or dermatosis, hair fragility, history of deep venous thrombosis (DVT), and neoplasia and participants with cardiac pacemaker or other implanted electronic devices.

## 3. Data Collection

The following methods of evaluation were used in this study: anthropometry for weight (kg) and height (m^2^) and calculation of BMI (kg/m^2^). Perimetry was performed for the evaluation of thigh circumference. For the evaluation of cellulite, the scale of severity of cellulite (Cellulite Severity Scale-CSS) was used. For the evaluation of skin elasticity, the Cutometer® MPA 580 (Courage-Khazaka, Germany) was used.

The analysis of the subcutaneous tissue thickness was performed by an ultrasonographer physician using diagnostic ultrasound equipment with the frequency of 6-18 MHz (MyLab ™ 25 Gold, Esaote, Italy) with software VPan (Esaote, Italy). The evaluation points are determined and marked by the main evaluator in the gluteal and posterior thigh regions.

For the evaluation of quality of life, the Celluqol® questionnaire, which evaluates how much the cellulite affects the quality of life and lifestyle of women, was applied. The questionnaire consists of questions related to the choice of clothes, diet, recreation, physical activities, relationship with the partner, feelings, and change of daily habits. Each question is scored 1 point (when the individual does not feel bothered by the fact of having cellulite) up to 5 (when the fact of having cellulite bothers the individual all the time). The final result of Celluqol® results from the sum of the points of each question.

The evaluations were carried out by a specialized physiotherapist, and an initial evaluation, an evaluation with six and ten treatment sessions, and an evaluation with a follow-up of 3 months after the end of treatment were performed.

## 4. Treatment Protocol

Ten sessions of ESWT were carried out, using the THORK® Shock Wave (IBRAMED-Indústria Brasileira de Equipamentos Médicos EIRELI, Brazil) equipment, twice a week, in the gluteal and posterior thigh region. The protocol determined was of 4 thousand shots with a radial stainless-steel tip of 15 mm of diameter, with energy of 200mJ, followed by 2 thousand shots with the radial polyacetal tip of 15 mm of diameter with energy of 100mJ. The frequency of shots was of 15 Hz and a neutral local lotion (Loção Neutra Thork®, Essencial Cosméticos, Brazil) was used as a contact medium and aide for the sliding of the tip.

## 5. Statistical Analysis

All data were presented in terms of mean and standard deviation. A normality test Lilliefors's was applied to verify the normal distribution of the samples (P-value > 0.05). The statistical difference between groups was determined by one-way ANOVA test (P-value < 0.05) and post hoc Tukey test to comparing the data (P-value < 0.05).

## 6. Results

Thirty women started the treatment; however, three of them were excluded due to absence in the sessions and reevaluations. Twenty-seven women completed the treatment. In the initial evaluation and reevaluations, the participants presented an average age of 33 ± 5 years, average weight of 65 ±10kg, maximum height of 1.62 ±0.06 meters, and average BMI of 24 ±3 kg/m^2^. There were no considerable alterations in weight and BMI during and after the treatment.

The measurements of perimetry and the analysis of thickness of the subcutaneous tissue did not present significant alterations from p< 0.05.

**Figure 1:**
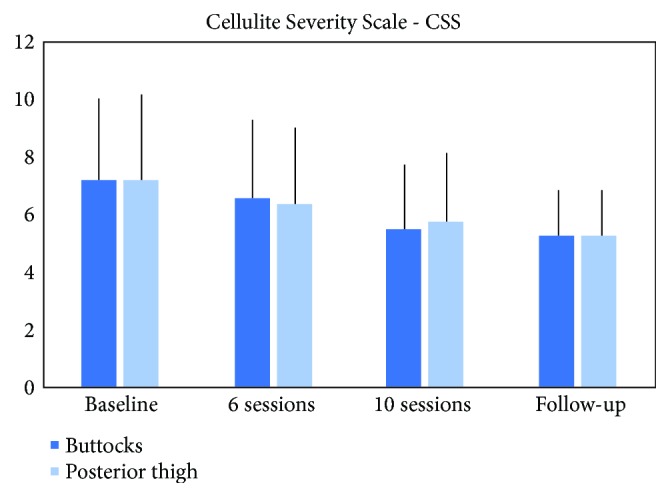
Improvement in the severity of cellulite between the initial evaluation, reevaluations, and follow-up of 3 months from the end of the treatment.

**Figure 2:**
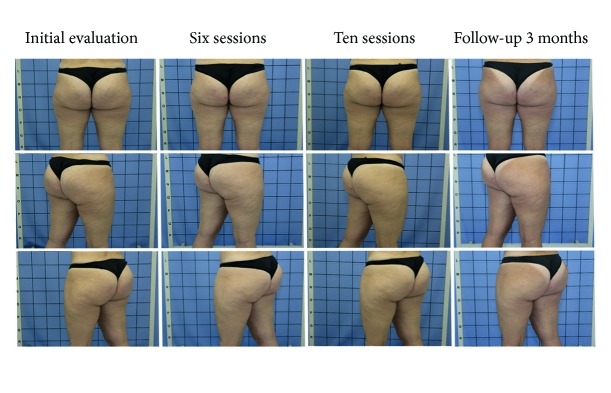
Improvement of the degrees of cellulite in different views in the comparison of the photos throughout the evaluations and reevaluations.

The analysis of the variables of CSS (Cellulite Severity Scale) demonstrated that there was significant improvement in the severity of cellulite between the initial evaluation, reevaluations, and follow-up of 3 months from the end of the treatment for gluteus (p <0.0087), being the average measurements (7.2±2.8 to 6.6±2.7 to 5.5±2.2 to 5.3±1.6) and posterior thigh (p < 0.0031), with average scores of 7.2±2.9 to 6.4 ±2.6 to 5.7±2.3 to 5.2±1.5, as seen in Figures 1 and 2.

The analyses of the Cutometer® data demonstrated significant results for the variable R2, which indicates the gross elasticity of the skin, being the value of p<0.05 in the comparison between the initial evaluation and the follow-up of 3 months and six sessions for the follow-up of 3 months, similarly, for the variable R8, which indicates the ability of skin deformation, that is, the ability of the skin to return to its initial state, being the value of p<0.05 between the reevaluation of six sessions and the follow-up of 3 months.

**Figure 3:**
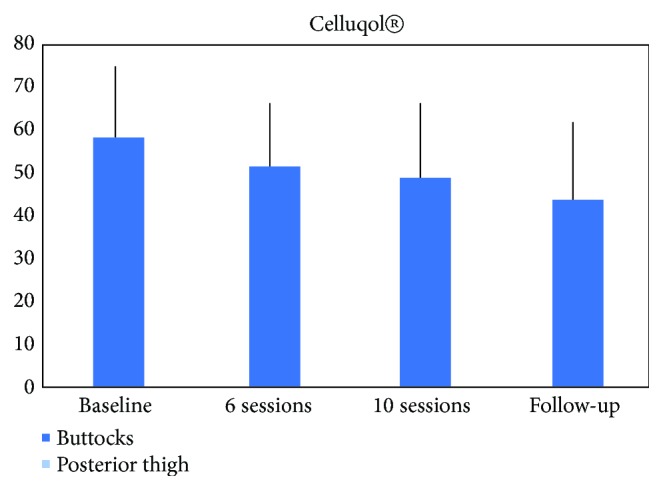
Improvement of quality of life by the representation of the Celluqol® questionnaire.

At the end of all the evaluations, the self-evaluation Celluqol® questionnaire was delivered to the participants, and it demonstrated significant results, especially between the initial evaluation and the follow-up of 3 months from the end of treatment, being the scores, respectively (*58±16* to 52±14 to 48±16 to* 45±16*= < 0.05), as presented in Figure 3.

During and after the treatment adverse reactions to the extracorporeal shockwave therapy were not found or related.

## 7. Discussion

Several treatment modalities and therapy associations were developed for the treatment of cellulite; however, science has been demonstrating that cellulite consists of a multifactorial metabolic disorder and, to a certain degree, enigmatic. Consequently, until the present moment there is no revolutionary therapy that will bring the cure for cellulite [12].

Nevertheless, technological evolution has been providing new resources for the reestablishment and improvement of health. ESWT is considered a relatively new technology in esthetic medicine which has a complex therapeutic objective that is treating cellulite demonstrating expressive results; this disorder, which bothers women to such a high extent, however, is not easy to be treated [9, 13].

It is demonstrated in the present study that although cellulite is a complex unaesthetic disorder, shockwave therapy obtained satisfactory results in the improvement of the degrees of severity of cellulite, morphological aspects of the skin, and quality of life.

This is the second published study which used the CSS scale to evaluate the results of ESWT. It was observed that regardless of the treatment area, there was reduction in the severity of cellulite through the CSS with significance of p< 0.05 for the degree of severity of cellulite presented during and after the end of 3 months of treatment; that is, the volunteers classified with severe degree of severity presented reduction to the moderate degree and the volunteers classified with the moderate degree were reclassified with light cellulite degree [8].

The present result corroborates the first article published about ESWT therapy in 2005, in which the researchers compared the independent application of ESWT and the combined form, with decongestive therapy for two weeks. The authors came to the conclusion that one single independent application of ESWT can improve the aspect of the cellulite, because the therapy can significantly decrease the levels of Plasma Malondialdehyde (MDA), a biomarker of the level of oxidative stress [14].

One of the elements which characterizes cellulite is the oxidative stress of the adipose tissue, potentialized by the level of inflammation and consequent level of tissue hypoxia, causing unbalance in the production and buffering of the free radicals [15].

Some studies report treatments which can be used to reduce the oxidative stress of cellulite, among them carboxytherapy, which favors vasodilation and tissue oxygenation with consequent increase of local and decrease of oxidative stress, and such results of this study and according to the Siemes et al. (2005) study, it is suggested that ESWT therapy can also play a role in the decrease of oxidative stress [16, 17].

Another study which confirms the findings is Hexsel et al.'s study (2016), in which the authors evaluated thirty-five women who underwent the treatment with ESWT in the gluteus and posterior thighs. The therapy demonstrated a positive effect of the degrees of severity of cellulite in the global sample up to 12 weeks from the end of the treatment. Some authors suggest that the improvement of the aspect of the cellulite through ESWT therapy is related to the improvement of local blood circulation, which interrupt the vicious cycle of the inflammatory process caused by the decrease of microcirculation of blood and lymph, generating the reorganization of the extracellular medium [8, 11, 13].

In the present study the significant result with p<0.05 was demonstrated in gross elasticity, when the variable R2 reached the value close to 1. Besides that, the ability of skin deformation also presented a significant result, when the variable R8 was closer to the value zero.

Out of eleven published articles about the role of ESWT in the treatment of cellulite, eight of them demonstrated improvement in the aspect of the skin, with increase in skin elasticity and firmness by histological improvement of the dermis and epidermis, extracellular matrix, and consequent collagen remodeling [18, 19].

It is know that ESWT acts on the biological tissue by means of mechanoreduction; that is, it exerts a mechanical stress on the skin which is converted in response at the tissue cellular level, stimulating the fibroblasts. The cells notice the changes in their environment in the extracellular matrix and convert the mechanical information in stimuli for the production of cellular growth factor, neocollagenesis, and neoelastogenesis [13, 20, 21].

According to Modena et al. (2017) “the main effects observed in the biological tissue, cause damage to the extracellular matrix, resulting in a cascade of physiological reactions which benefit the reorganization of the extracellular medium. These effects also include the increase of blood and lymphatic circulation, modifications in the permeability of the cellular membrane and contribute in the liberation of nitric oxide. Subsequently, there is balance in the production of free radicals, increase in the capability of drainage of molecular proteins”. Other results of the action of ESWT on biological tissue suggest that ESWT may induce lipolysis and/or apoptosis of the adipose cell; however, little is known about which is the real mechanism of action of the therapy.

It is worth highlighting that, besides the improvement in the mechanical properties of the skin during the treatment, this benefit was maintained for three months after the end of the treatment. This demonstrates that the stimulus of ESWT on the fibroblasts continues even after the end of the therapy. This result reaffirms the data found by Angehrn et al. (2007) who evaluated the effect of ESWT in the treatment of cellulite in the lateral region of thighs, in 21 women. They related that there was improvement of cellulite by collagen remodeling and realignment of dermal fibers and that these effects remained latent six months after the end of the treatment [22].

Another important variable to be evaluated is the quality of life of women suffering from cellulite, and for that purpose the Celluqol® questionnaire was used. This study demonstrated that the quality of life of the participants in the research was getting better during the treatment and remained even 3 months after the end of the treatment [19].

It is well known that health does not represent only physical well-being or solely the absence of disease; it is also the inability of the individual to have a productive life, both personally and professionally [1, 23].

Dissatisfaction with one's body reflects in the several concepts related to beauty standards that each culture develops, and consequently the body image is influenced by different sociocultural, biological, and environmental aspects which generate conditions of depression, anxiety, and low self-esteem which so much affect women nowadays [23].

ESWT demonstrated to be an effective and safe therapy in the treatment of the cellulite condition, aspect of the skin, and in particular in the improvement of quality of life, corroborating other previously published studies with the same treatment objective and highlighting that the treatment with this therapeutic modality can indeed contribute to new technological advances towards the treatment of cellulite [9, 13].

## 8. Conclusion

It can be concluded that ESWT with a magnetic generator and applicator by radial waves is efficient and safe in the clinical treatment of cellulite, with the objective of decreasing its degrees, improving the aspect of the skin, and reestablishing quality of life with lasting results even after the end of the treatment.

## Data Availability

The data used to support the findings of this study are available from the corresponding author upon request.
